# Quantification of the area-at-risk by T1 and T2 mapping CMR at 3T

**DOI:** 10.1186/1532-429X-17-S1-P8

**Published:** 2015-02-03

**Authors:** Heerajnarain Bulluck, Steven K White, Stefania Rosmini, Anish N Bhuva, Georg M Frohlich, Thomas A Treibel, Marianna Fontana, Amna Abdel-Gadir, Anna S Herrey, Charlotte Manisty, Ming Young S Wan, Ashley Groves, Leon J Menezes, Peter J Weale, James Moon, Derek J Hausenloy

**Affiliations:** 1 The Heart Hospital/ University College London Hospital, London, UK; 2 The Hatter Cardiovascular Institute, University College London, London, UK; 3 University College London Hospital, London, UK; 4 Siemens Healthcare, Frimley, UK

## Background

Measuring the area-at-risk (AAR) allows the assessment of myocardial salvage in reperfused STEMI patients. T2-weighted CMR has been used to quantify the AAR but is hampered by low signal-to-noise ratio and image artifacts. T1 and T2 mapping CMR may improve upon this. We compared T1 and T2 mapping for quantifying the AAR at 3T.

## Methods

**Figure 1:**
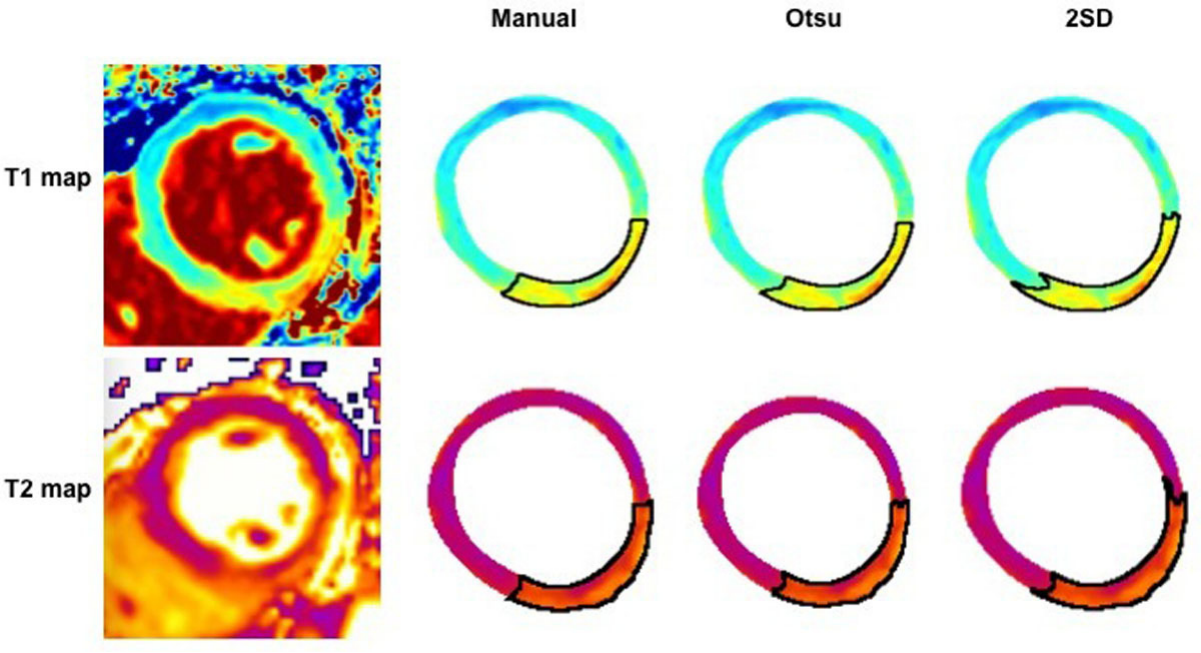
Mid-ventricular short-axis slice of a patient with a reperfused inferior STEMI depicting the area-at-risk assessed by the manual, Otsu and 2SD threshold techniques using ImageJ

CMR imaging at 3T (Bio-graph mMR, Siemens Healthcare, Erlangen, Germany) was performed in 18 STEMI patients within 10 days of PPCI using T2-mapping and T1-mapping by MOLLI (WIP #699; Siemens Healthcare; UK). Matched short-axis T1 and T2 maps covering the entire left ventricle were analyzed by 2 experienced investigators using 3 analytical methods (in-house macro, ImageJ): manual segmentation, Otsu, and the 2 standard deviation (2SD) thresholding (Fig. 1). Regions-of-interest were drawn in the AAR and remote myocardium. Two investigators analyzed the coronary angiograms to obtain the BARI and APPROACH angiography scores to provide a CMR-independent estimate of the AAR.

## Results

**Figure 2:**
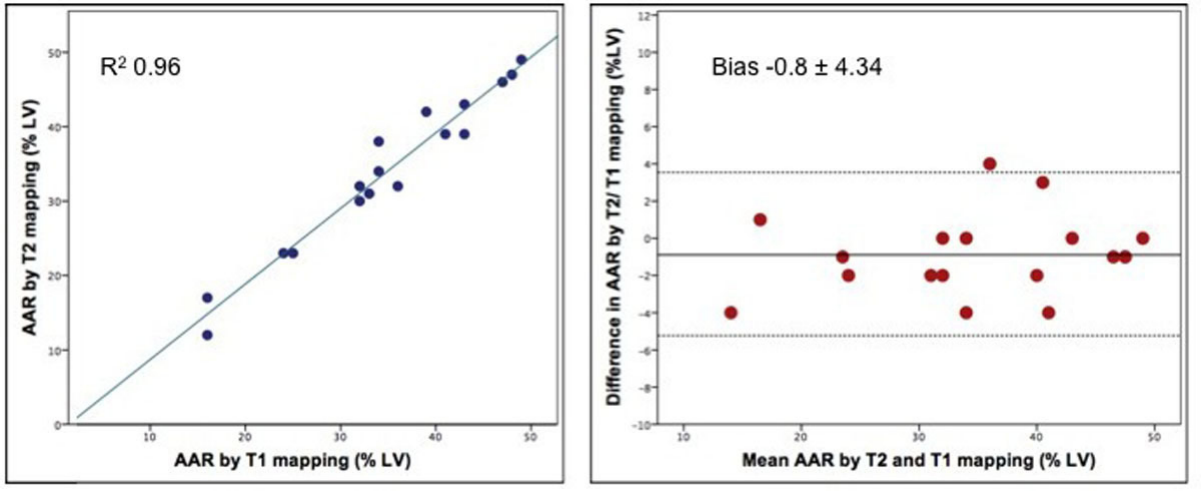
There is excellent correlation and agreement between the area-at-risk quantified by T2 and T1 mapping CMR (Otsu threshold technique)

T1 and T2 values were increased within the AAR compared with remote myocardium (mean±SD: T1, 1525±116ms vs. 1163±78ms, P<0.001 and T2, 72±7ms vs. 46±3ms, P<0.001). Analysis of the T1 and T2 maps using the manual and Otsu techniques yielded similar results for the AAR, whereas the AAR quantified by the 2SD technique was about 7% larger. There was excellent inter-observer variability for T1 and T2 mapping using all 3 analytical techniques (Intraclass Correlation Coefficient>0.98:P<0.001). The Otsu-derived AAR (expressed as a % of the left ventricle [LV] volume) quantified by T1 and T2 mapping were similar (35.6±10.3% vs. 34.7±10.7%; P>0.05). There was excellent correlation (R^2^=0.96: P<0.001; Fig. 2) and agreement (Bias -0.8±4.34%; Fig. 2) between the AAR quantified by T1 and T2 mapping. Finally, the AAR delineated by both T1 and T2 mapping correlated with the BARI (T1: R=0.85, P<0.001; T2: R=0.81,P<0.001) and APPROACH (T1: R=0.82, P<0.001; T2: R=0.79, P<0.001), although the CMR-derived AAR measurements were significantly larger.

## Conclusions

T1 and T2 mapping CMR can accurately quantify the AAR at 3T in reperfused STEMI patients. Although both mapping techniques yielded similar results, more work needs to be done to see if T1 mapping has any benefit over T2 mapping in patients with very short onset to balloon time; stability of the signal up to 2 weeks post myocardial infarction and whether therapies reducing edema by T2 mapping also affects T1 mapping.

## Funding

This work was supported by the British Heart Foundation (FS/10/039/28270;FS/10/72/28568), the Rosetrees Trust, and the National Institute for Health Research University College London Hospitals Biomedical Research Centre.

